# Recombinant MAM from *Faecalibacterium duncaniae* exhibits a protective effect in DNBS-induced colitis

**DOI:** 10.1186/s12934-025-02877-9

**Published:** 2025-12-06

**Authors:** Thaís Vilela Rodrigues, Luís Lima de Jesus, Monique Ferrary Américo, Florian Chain, Laura Creusot, Nathalie Rolhion, Anne Aucouturier, Luis Bermudez-Humaran, Philippe Langella, Vasco Ariston de Carvalho Azevedo, Jean-Marc Chatel

**Affiliations:** 1 Instituto de Ciências Biológicas, Federal University of Minas Gerais, Belo Horizonte, Minas Gerais Brazil; 2 INRAE, AgroParisTech, MICALIS, Université Paris Saclay, Jouy-en-Josas, France; 3 Inserm, Centre de Recherche Saint-Antoine, CRSA, Sorbonne Université, Paris, 75012 France; 4 Gut, Liver & Microbiome Research FHU, Paris, France

**Keywords:** Inflammatory bowel disease, Next-generation probiotic, Peptide, Effector molecule, Purification

## Abstract

**Background:**

Microbial anti-inflammatory molecule (MAM) is a key effector of the next-generation probiotic *Faecalibacterium duncaniae* A2-165, a species whose depletion in the gut microbiota is strongly linked to inflammatory bowel disease (IBD) and other conditions. Despite its importance, the direct anti-inflammatory effects of purified MAM have never been evaluated in vitro or in intestinal inflammation models. Prior studies have relied on bacterial supernatants, synthetic peptides, or DNA delivery systems, each with inherent limitations.

**Results:**

In this study, we produced and purified recombinant MAM (R-MAM) under denaturing conditions and, for the first time, demonstrated its direct anti-inflammatory activity in vitro and its protective effects in a colitis murine model. Despite numerous attempts, we were not able to obtain a non-aggregated R-MAM. Therefore, we can assume that the R-MAM used here is partly or totally denatured. Nevertheless, in vitro assays with human intestinal epithelial cells (HT-29) and peripheral blood mononuclear cells (PBMCs) confirmed the ability of MAM to induce an anti-inflammatory cytokine profile. In addition, in a DNBS-induced colitis model, oral administration of R-MAM significantly prevented weight loss and reduced colon weight and thickness, key macroscopic indicators of inflammation.

**Conclusions:**

These findings provide a critical validation step for the therapeutic potential of MAM in intestinal inflammation, despite its purification under denaturing conditions. Future studies should focus on optimizing protein stability and conformational integrity to increase its therapeutic potential as a biotherapeutic agent.

**Graphical Abstract:**

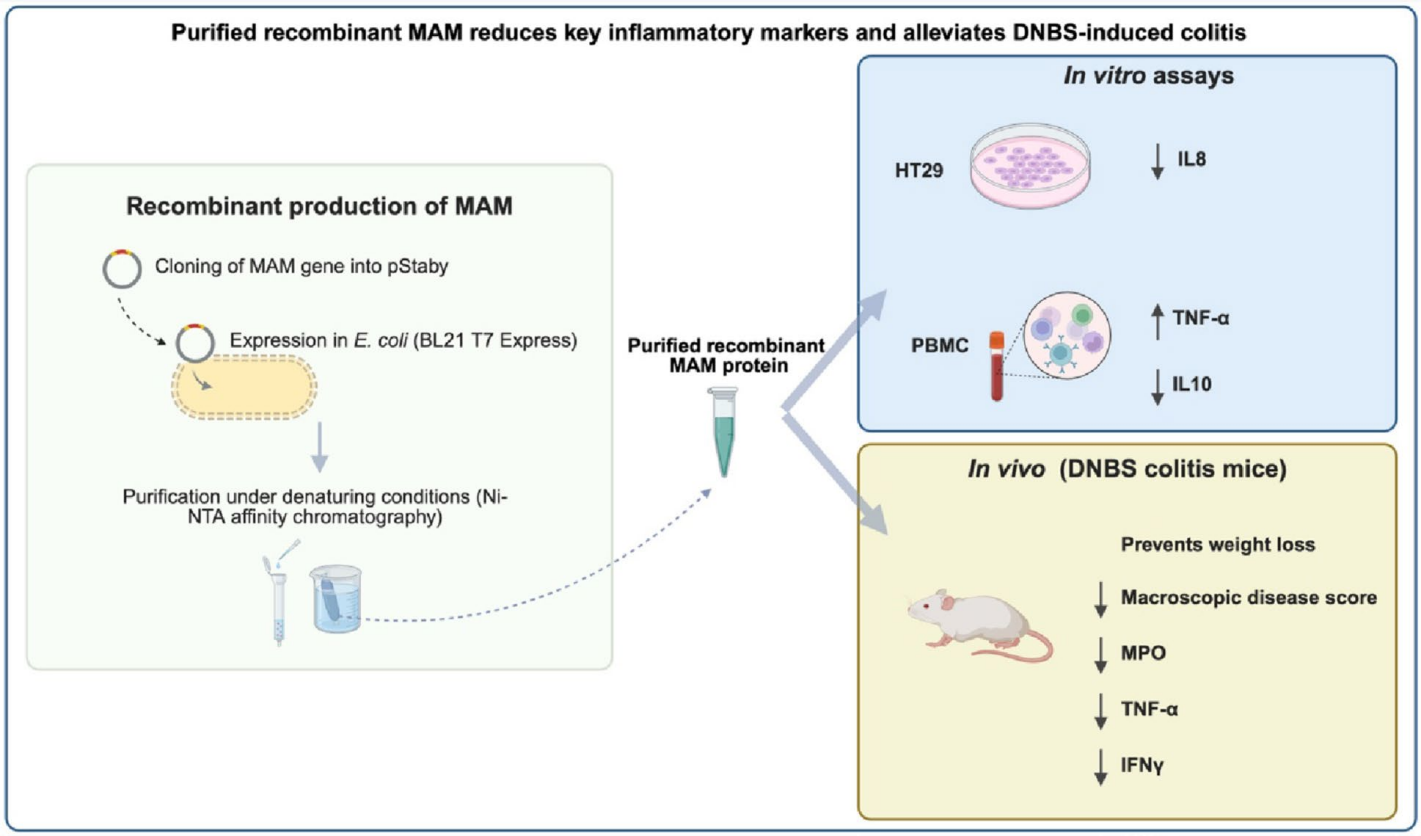

**Supplementary Information:**

The online version contains supplementary material available at 10.1186/s12934-025-02877-9.

## Background

Inflammatory bowel diseases are chronic disorders of the gastrointestinal tract that result from a complex interplay between genetic, immune, microbial, and environmental factors [1–3]. This condition includes Crohn’s disease (CD) and ulcerative colitis (UC). A central aspect of IBD pathogenesis is the imbalance in cytokine signaling, where excessive pro-inflammatory mediators, such as TNF-α, IL6, and IL1β, drive chronic inflammation and tissue damage. This process is further exacerbated by gut dysbiosis. While targeted biologic therapies such as anti-TNF, anti-IL12/23, and integrin inhibitors have improved disease management, the therapeutic response remains highly variable, emphasizing the need for precision medicine approaches based on molecular and microbiota-based profiling [4, 5].

A key marker of IBD patients is the significant depletion of the *F. duncaniae* (previously named *F. prausnitzii*) strain A2-165 within the gut microbiome [6–9]. *F. duncaniae* is an extremely oxygen-sensitive Bacillus belonging to the Bacillota phylum (formerly Firmicutes) that constitutes a substantial proportion of the gut microbiota. This genus represents rates ranging from 5 to 15% of the bacterial community composition [8, 10]. This abundance is associated with a healthy gut environment, whereas reduced levels of *F. duncaniae* have been correlated with IBD and various disorders, including colorectal cancer, diabetes, and obesity [11–16].

Several beneficial properties are attributed to *F. duncaniae*, such as anti-inflammatory properties, reinforcement of the intestinal barrier, and maintenance of gut homeostasis [17–19]. This activity is attributed mainly to its remarkable capacity for butyrate production and its exclusive ability to synthesize MAM [18, 19]. MAM is a 15 kDa protein with potent anti-inflammatory properties that was first identified in the supernatant of *F. duncaniae* [20, 21]. Using cDNA delivery systems, MAM has been shown to modulate the NF-κB signaling pathway and reduce pro-inflammatory cytokine secretion in vitro and in vivo, particularly in colitis models [19, 22, 23]. However, these approaches, while demonstrating the biological activity, rely on indirect expression systems that introduce variability in protein availability and function.

Despite its promising anti-inflammatory effects, the therapeutic potential of purified recombinant MAM (R-MAM) remains largely unexplored. To date, only one study has evaluated purified R-MAM, which showed benefits in a diabetes mellitus (DM) model, particularly in enhancing intestinal barrier integrity [24]. However, its direct effects in vivo in intestinal inflammation models have not yet been assessed. This study aims to address this gap by investigating the impact of purified R-MAM in both in vitro models (HT-29 cells and PBMCs) and in a DNBS-induced colitis mouse model. Despite the fact that R-MAM is probably denatured, by using purified R-MAM, we reduce the bias associated with cDNA-based expression systems and enable a more controlled and direct assessment of its therapeutic potential, providing new insights into R-MAM activity in intestinal inflammation.

## Methods

### R-MAM expression induction

The coding sequence of the MAM from *F. duncaniae* A2-165 (WP_005932151) was inserted into the pStaby vector with restriction enzymes (Xhol and Nhel). The resulting recombinant plasmid was transformed into chemically competent *Escherichia coli* T7 Express (BL21) for protein expression. A single colony was inoculated into 10 mL of Luria–Bertani (LB) medium supplemented with 100 µg/mL ampicillin and incubated overnight at 37 °C with shaking (180 rpm). Subsequently, 5 mL of the overnight culture was transferred into 500 mL of LB medium containing 100 µg/mL ampicillin. When the culture reached an optical density at 600 nm (OD600) of 0.6–0.8, the expression of recombinant MAM (R-MAM) was induced by adding 1 mM isopropyl β-D‐thiogalactoside (IPTG), followed by incubation at 37 °C for 19 h with shaking (180 rpm). The cells were then harvested via centrifugation (2500×*g* for 10 min at 4 °C), washed twice with ice-cold 0.1 M phosphate-buffered saline (PBS; pH 7.0), and centrifuged again under the same conditions. The resulting bacterial pellets were stored at − 20 °C for a maximum period of 24 h until protein extraction.

### Extraction and purification of R-MAM under denaturing conditions

A maximum of four bacterial pellets, each obtained from 125 mL of culture, were utilized for protein extraction and purification. The frozen pellets were thawed and subsequently resuspended in Buffer B (8 M urea, 100 mM NaH2PO4, 10 mM Tris, and 50 mM imidazole; pH 8.0) at a ratio of 15 mL of buffer per pellet. The suspensions were incubated at room temperature for 1 h, with intermittent homogenization every 20 min. Following incubation, the samples were sonicated on ice for 4 cycles of 20 s each. The homogenate was then centrifuged at 10,000×*g* for 25 min at 21 °C to remove insoluble debris. The resulting supernatant was carefully collected and loaded onto a Ni-NTA resin column (1.5 mL) (Invitrogen) preequilibrated with Buffer B.

The column was sequentially washed twice with 5 mL of Buffer C (8 M urea, 100 mM NaH2PO4, 10 mM Tris, 50 mM imidazole, and 500 mM NaCl; pH 6.3), once with Buffer D1 (8 M urea, 100 mM NaH2PO4, 10 mM Tris, 50 mM imidazole, and 200 mM NaCl; pH 5.9), and finally once with Buffer D2 (8 M urea, 100 mM NaH2PO4, 10 mM Tris, 50 mM imidazole, and 300 mM NaCl; pH 5.3). Following the washing steps, the purified MAM was eluted in 500 µL fractions via Buffer E (8 M urea, 100 mM NaH2PO4, and 10 mM Tris; pH 4.2). To optimize purity, this step was performed at pH 4.2 instead of the recommended pH of 4.5, as a lower pH improved the specificity of MAM recovery by reducing the coelution of contaminant proteins. Aliquots (20 µL) of the eluted fractions were visualized via SDS‒PAGE.

The purification procedures were adapted from the Expression and Purification of Proteins via the 6x Histidine-Tag Manual (https://www.iba-lifesciences.com/media/a8/ee/aa/1631860506/Manual-6xHistidine-tag.pdf), with modifications to optimize protein yield and purity.

### R-MAM protein dialysis

Dialysis of the purified R-MAM protein was conducted via a stepwise protocol to gradually reduce the urea concentration. Approximately 5 mL of purified protein (~ 2.5 mg/mL) was loaded into a 10 kDa molecular weight cutoff dialysis cassette (Spectra/Por^®^ 7 Dialysis Membrane). The cassette was sequentially dialyzed against 200 mL of buffers with decreasing urea concentrations as follows: Buffer 1 (6 M urea, 50 mM Tris, 1% glycerol, and 200 mM NaCl; pH 7.4) for 1 h, followed by Buffer 2 (4 M urea, 50 mM Tris, 1% glycerol, and 200 mM NaCl; pH 7.4); Buffer 3 (2 M urea, 50 mM Tris, 1% glycerol, and 200 mM NaCl; pH 7.4); Buffer 4 (1 M urea, 50 mM Tris, 1% glycerol, and 200 mM NaCl; pH 7.4); and Buffer 5 (0.1 M PBS containing 1% glycerol; pH 7.4). After 1 h of dialysis in Buffer 5, the buffer was replaced twice, and the dialysis process was continued for an additional 24 h. The dialyzed protein mixture was then collected directly from the cassette (Supplementary Fig. 1), analyzed via SDS‒PAGE, quantified via bicinchoninic acid (BCA) assay (Thermo Fisher), and stored at − 80 °C for subsequent in vitro and in vivo assays.

### *F. duncaniae* culture preparation

The strain *Faecalibacterium duncaniae* A2-165 (DSM #17677) was cultivated in BHIS liquid medium (Brain Heart Infusion broth, 37 g/L, Difco) supplemented with yeast extract (5 g/L, Difco) and 3% GAC (glucose, acetate, cysteine), a mixture composed of 6.6% glucose, 5.5% acetate, and 1.66% cysteine. GAC supplementation was used to support bacterial growth, where acetate serves as the main energy source, cysteine protects against oxygen, and glucose provides additional metabolic support. The cultures were incubated at 37 °C in an anaerobic chamber under an atmosphere consisting of 90% nitrogen (N₂), 5% carbon dioxide (CO₂), and 5% hydrogen (H₂). Bacterial growth was monitored until the cultures reached the stationary phase, as indicated by an optical density at 600 nm (OD₆₀₀) of approximately 1.6–1.7. The samples were collected at this point and stored at − 80 °C with 20% glycerol for future use.

### HT-29 culture and in vitro anti-inflammatory assay

Human colon carcinoma cells (HT-29 ATCC HTB-38) were grown in Dulbecco’s modified Eagle’s minimal essential medium (DMEM) with 4.5 g/L glucose (Sigma‒Aldrich) supplemented with 10% (v/v) heat-inactivated fetal bovine serum (FCS; Eurobio, France) and 0.1% penicillin/streptomycin (P/S). The cultures were maintained at 37 °C in a 10% (v/v) CO2 atmosphere until 100% confluence, and the medium was replaced every 2 days.

For an anti-inflammatory assay with the HT-29 cell line, 1 × 105 cells were seeded in 24-well plates and cultivated in DMEM supplemented with 10% FBS and 0.1% P/S. On the 6th day after seeding, the medium was changed to DMEM supplemented with 5% FBS and 0.1% P/S. Co-incubation started on the same day, with the cells at confluence. The cells were stimulated for 24 h at 37 °C in 10% CO2 with recombinant TNF-α (5 ng/mL; PeproTech, NJ, USA) to induce a pro-inflammatory state and simultaneously co-incubated with 50 µL of either recombinant MAM (R-MAM) at final concentrations of 0.05 mg/mL or 0.1 mg/mL, or 50 µL of PBS as the vehicle control. The experiments were conducted in triplicate following standardized conditions in the protocols established by our facility [25–27]. The supernatants were collected and used for IL8 concentration measurement via ELISA (BioLegend, San Diego, CA). The procedures were performed according to the manufacturer’s instructions.

### PBMC in vitro anti-inflammatory assay

Fresh peripheral blood mononuclear cells (PBMCs) were obtained from four healthy donors. Approval for blood collection was obtained from the local ethics committee (Comité de Protection de Personnes Ile de France IV, IRB 00003835 Suivitheque study). Sepmate tubes (STEMCELL Technologies, 85450) were used for cell isolation via density gradient centrifugation, following the manufacturer’s instructions. PBMCs were resuspended in RPMI-1640 Glutamax (Gibco, 61870-010) supplemented with 10% FBS (Sigma #F7524), 1% P/S (Gibco, #15140-122), 1% pyruvate (Gibco, #11360-039), and 1% nonessential amino acids (Gibco, #11140-035), and 1 × 106 PBMCs per well were seeded. The cells were subsequently stimulated for 16 h with doses of MAM ranging from 0.5 to 0.001 mg/mL final concentration, and 1 µg/mL *E. coli* K12 lipopolysaccharide (LPS, InvivoGen, tlrl-eklps) was used as a positive control.

To account for the potential effects of cell viability on cytokine measurements, cytokine levels (IL10 (Invitrogen, #88-7106-88) and TNF-α (Invitrogen, #88-7346-88)) were normalized to lactate dehydrogenase (LDH) activity in the culture supernatant. LDH release, measured via a cytotoxicity detection kit (Roche, 04744934001), served as an indicator of cell lysis. Cytokine concentrations are expressed relative to LDH levels in the corresponding wells to adjust for variations in cell integrity. The experiments were conducted in duplicate.

### DNBS-induced colitis mouse model anti-inflammatory assay

The experiment was conducted according to the ethics committee guidelines. Six-week-old male C57BL/6JRj mice (Janvier, France) (*n* = 8 mice per group) were maintained in the animal facilities of the French National Institute of Agricultural Research (IERP; INRA Jouy-en-Josas, France) under specific pathogen-free (SPF) conditions. Animals were kept in plastic cages with 4 mice per cage under the temperature of 21 °C, with unrestricted access to food and water. A 12-h light/dark cycle was provided. To ensure acclimatization, the animals were maintained under these conditions for 1 week prior to the start of the experiments. Intragastric gavage was initiated on day 1 with 200 µL of *F. duncaniae* at a concentration of 1 × 10⁹ CFU/mL, recombinant MAM (R-MAM) at concentrations of 1.0 mg/mL, 0.1 mg/mL, or 0.01 mg/mL, or PBS containing 16% glycerol, used as vehicle control for both R-MAM and *F. duncaniae* cell resuspension. Doses of R-MAM were chosen on the basis of previous studies investigating similar bioactive proteins in murine models [20–22]. Treatments were administered daily for the duration of the assay. On the 8th day, the mice were anesthetized and subjected to intrarectal injections of DNBS (100 mg/kg of body weight) diluted in 30% ethanol to induce colitis. The body weights of the mice were monitored daily throughout the experiment, and the animals were euthanized 4 days after the induction of DNBS-induced colitis (Fig. 4A). Mice that exhibited weight loss of 20% due to disease severity were euthanized according to established criteria.

Macroscopic scores were calculated for each mouse on the basis of ulcerations (scored 0–5), adhesions (presence/absence: 0/1), hyperemia (presence/absence: 0/1), altered transit, such as diarrhea or constipation (presence/absence: 0/1), and increased colon wall thickness (presence/absence: 0/1), which was measured via an electronic caliper (Control Company, WVR, United States) [28]. The total macroscopic score was determined by summing the individual scores for each animal.

### Myeloperoxidase (MPO) activity

Colon samples (1 cm) from mice were collected, weighed, and stored at – 80 °C. Tissues were homogeneized in 500 µL of hexadecyl trimethyl ammonium bromide (HTAB) buffer (Sigma- Aldrich, USA), using a Precellys^®^ homogenizer coupled to a Cryolys^®^ Evolution cooling system (Ozyme, France) for three cycles of 30 s at 8000 rpm at 4 °C. After centrifugation (14,000×*g* for 5 min at 4 °C), the homogenates were collected for MPO activity measurement. For this assay, 7 µL of each supernatant was mixed with 200 µL o-Dianisidine solution containing 1% H 2 0 2 in a 96-well plate. After incubation at room temperature for 1 h, absorbance was measured in 450 nm using a spectrophotometer (TECAN, Switzerland). MPO activity was expressed as enzyme unit per gram of tissue (U/g) based on MPO standard curve (Sigma-Aldrich, USA).

### Colonic cytokine levels

Colon supernatants previously prepared for MPO activity measurement were also used to quantify cytokines IL10, IL17A, TNFα and IFNγ using the ELISA MAX™ Standart Set (Biolegend, USA), according to the manufacturer’s instructions.

### Lipocalin-2 levels

The concentrations of Lipocalin-2 (Mouse Lipocalin-2, R&D Systems, USA) was quantified by enzyme-linked immunosorbent assay (ELISA) in colon and stools of mice, following the manufacturer’s protocols.

### Statistical analysis

Prism version 8 (GraphPad Software, San Diego, USA) was used to plot the results. Data are presented as the mean ± standard deviation (SD). Normality was assessed using the Shapiro-Wilk test. Depending on the results of the normality test, data were analyzed using Dunn’s test, Dunnett’s multiple comparisons test, the Kruskal-Wallis test, or analysis of variance (ANOVA). The specific statistical tests and post-test analyses used are indicated in the figure legends. Significance levels are represented as follows *: *p* < 0.05, **: *p* < 0.01, ***: *p* < 0.001, ****: *p* < 0.0001.

## Results

### Recombinant MAM displays anti-inflammatory activity in vitro in HT-29 cells

**Fig. 1:**
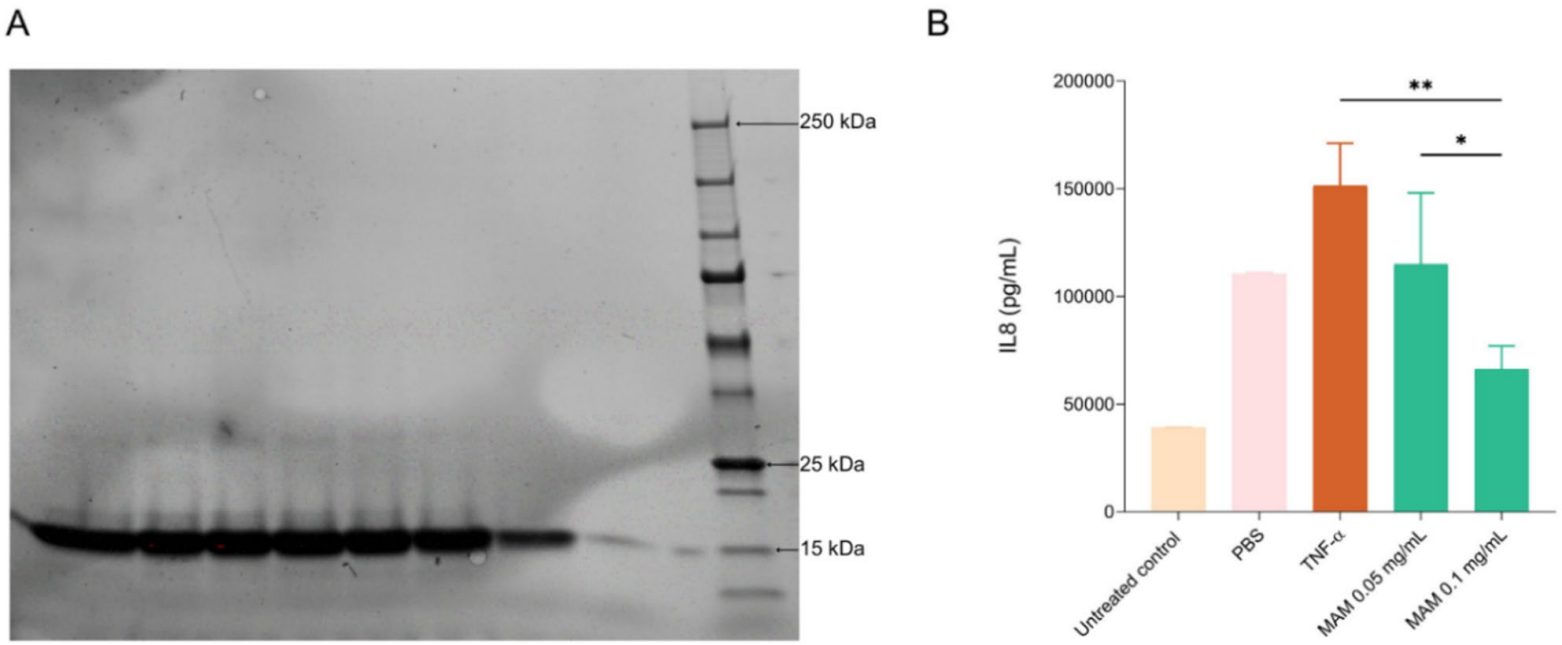
SDS-PAGE of purified mam and its effects on IL8 secretion in HT-29 cells. **a** SDS‒PAGE gel of purified MAM highlighting an intense band of MAM (expected size ~ 15 kDa) in all wells. **b** IL8 secretion by HT-29 cells subjected to inflammatory conditions by TNF-α. The treatment groups included TNF-α alone, TNF-α with PBS, and TNF-α with recombinant MAM (R-MAM) at concentrations of 0.05 mg/mL and 0.1 mg/mL. The beige and light pink bars represent the growth media for the HT-29 cells and the PBS controls, respectively. Statistical analysis was performed via ANOVA followed by Tukey’s multiple comparisons post test. Significant comparisons are indicated: *p* < 0.01 (**) and *p* < 0.05 (*)

After dialysis, the protein was obtained as aggregates, indicating probably a non-native folding. We used this insoluble form for our in vitro and in vivo assays (Fig. 1A and Supplementary Fig. 1).

The intestinal cell line HT-29 secreting IL8 in response to TNF-α stimulation was used to assess the immunomodulatory effects of R-MAM. Compared with TNF-α, R-MAM significantly reduced IL8 levels at the highest concentration tested (0.1 mg/mL) (*p* = 0.0012), resulting in a 56.32% reduction in IL8 secretion relative to that of the TNF-α-treated control (Fig. 1B). Interestingly, no significant difference was observed between the R-MAM 0.1 mg/mL group and the untreated control group (*p* = 0.3993), suggesting that this concentration tends to restore IL8 levels to basal levels. In contrast, a lower dose of R-MAM (0.05 mg/mL) tended to reduce IL8 levels (114,957 pg/mL vs. 151,423 pg/mL for TNF-α), although this reduction was not statistically significant (*p* = 0.1684). The greater reduction observed at 0.1 mg/mL supports a dose-dependent effect, with higher concentrations of R-MAM more effectively suppressing IL8 secretion. PBS, used as the vehicle for MAM, was also tested under TNF-α stimulation, and no significant difference in IL8 levels was observed compared with the TNF-α-stimulated control.

### Recombinant MAM induces IL10 and TNF-α production by PBMCs in a dose-dependent manner

**Fig. 2:**
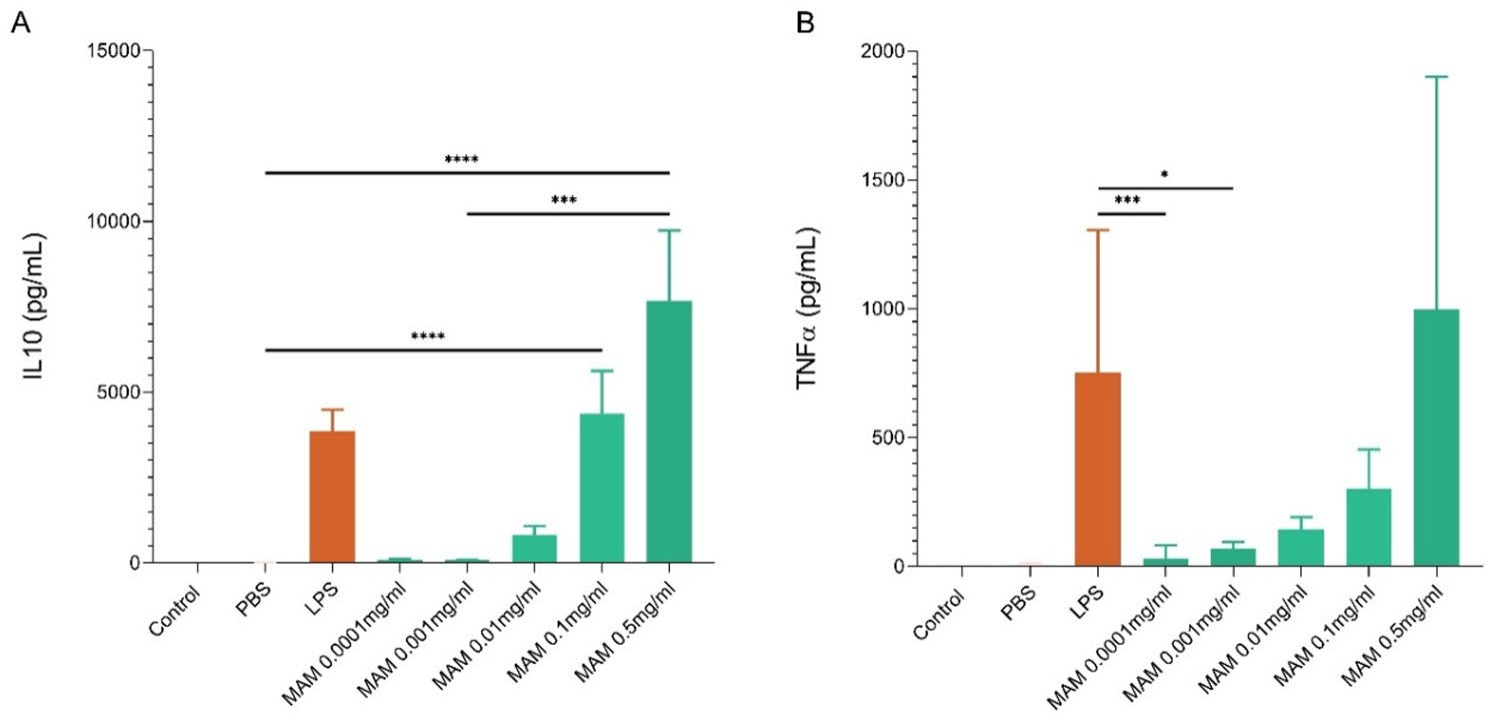
Quantification of IL10 and TNF-α production by PBMCs stimulated with R-MAM. **a** Quantification of IL10 and **b** TNF-α production by PBMCs stimulated with R-MAM via ELISA. The beige and light pink bars, which represent the growth media for the PBMCs and PBS controls, respectively, are not visually discernible due to their minimal values. The orange bars represent the LPS-stimulated control, whereas the green bars indicate the responses to different doses of R-MAM. Statistical analysis was performed via the Kruskal‒Wallis test followed by Dunn’s multiple comparisons post-test. Significant comparisons are indicated: *p* < 0.0001 (****), *p* < 0.001 (***), and *p* < 0.05 (*)

To evaluate the anti-inflammatory properties of MAM, PBMCs isolated from four healthy donors were stimulated with R-MAM at five different concentrations ranging from 0.5 to 0.0001 mg/mL. LPS (100 ng/mL) was used as a positive control, and PBS was used as a negative control. After 16 h of stimulation, R-MAM significantly induced IL10 secretion in a dose-dependent manner. Higher concentrations of R-MAM (0.5 mg/mL and 0.1 mg/mL) induced significantly greater IL10 than did PBS (*p* < 0.0001), although the difference was not significant compared with that of LPS (*p* > 0.9999) (Fig. 2A). Compared with LPS, lower concentrations of R-MAM (0.001 mg/mL and 0.0001 mg/mL) resulted in significantly lower TNF-α levels, whereas higher doses, such as 0.1 mg/mL R-MAM, did not significantly differ from those of LPS (*p* > 0.05), resulting in higher TNF-α levels (Fig. 2B).

**Fig. 3:**
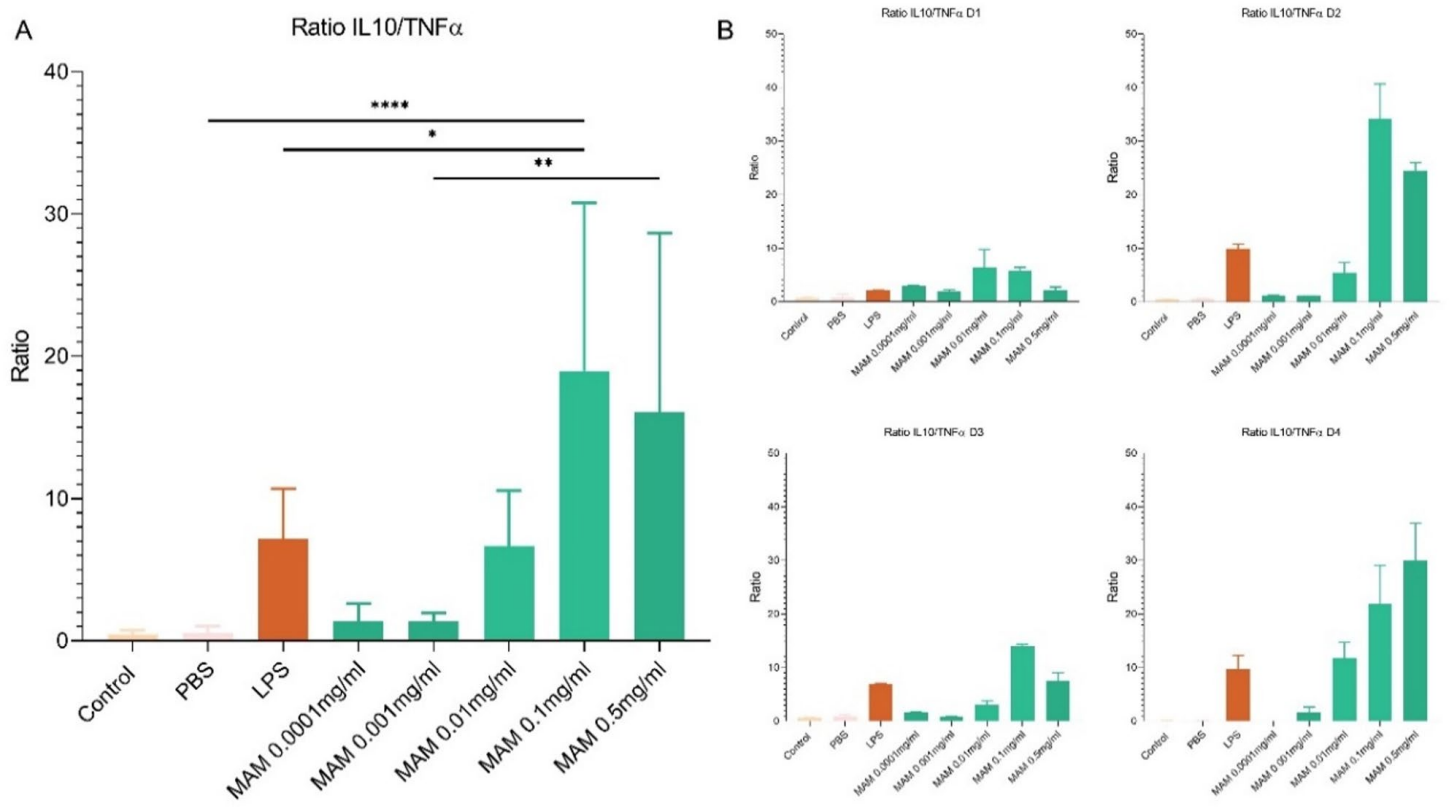
IL-10/TNF-α ratio in PBMCs stimulated with R-MAM. **a** The global panel shows the mean IL10/TNF-α ratio across all donors, whereas the donor-specific panels **b** illustrate individual responses to R-MAM stimulation at concentrations ranging from 0.5 to 0.0001 mg/mL (green bars). LPS was used as an inflammatory control. Ratios were calculated on the basis of cytokine levels quantified via ELISA. Statistical analysis was performed via ANOVA followed by Tukey’s multiple comparisons post-test. Significant comparisons are indicated: *p* < 0.0001 (****), *p* < 0.01 (**) and *p* < 0.05 (*)

To better evaluate the anti-inflammatory properties of R-MAM, we expressed the previous results as the IL10/TNF-α ratio, which indicates the balance between anti-inflammatory (IL10) and pro-inflammatory (TNF-α) responses. Notably, R-MAM at a concentration of 0.1 mg/mL had a significantly greater ratio than did LPS (*p* < 0.05), supporting its anti-inflammatory profile. The effect diminished with lower concentrations of R-MAM, which induced ratios similar to those of the PBS or LPS controls (Fig. 3A). Interestingly, the individual responses among donors varied, as shown in the donor-specific graphs (Fig. 3B).

### R-MAM alleviates DNBS-induced colitis in mice

**Fig. 4:**
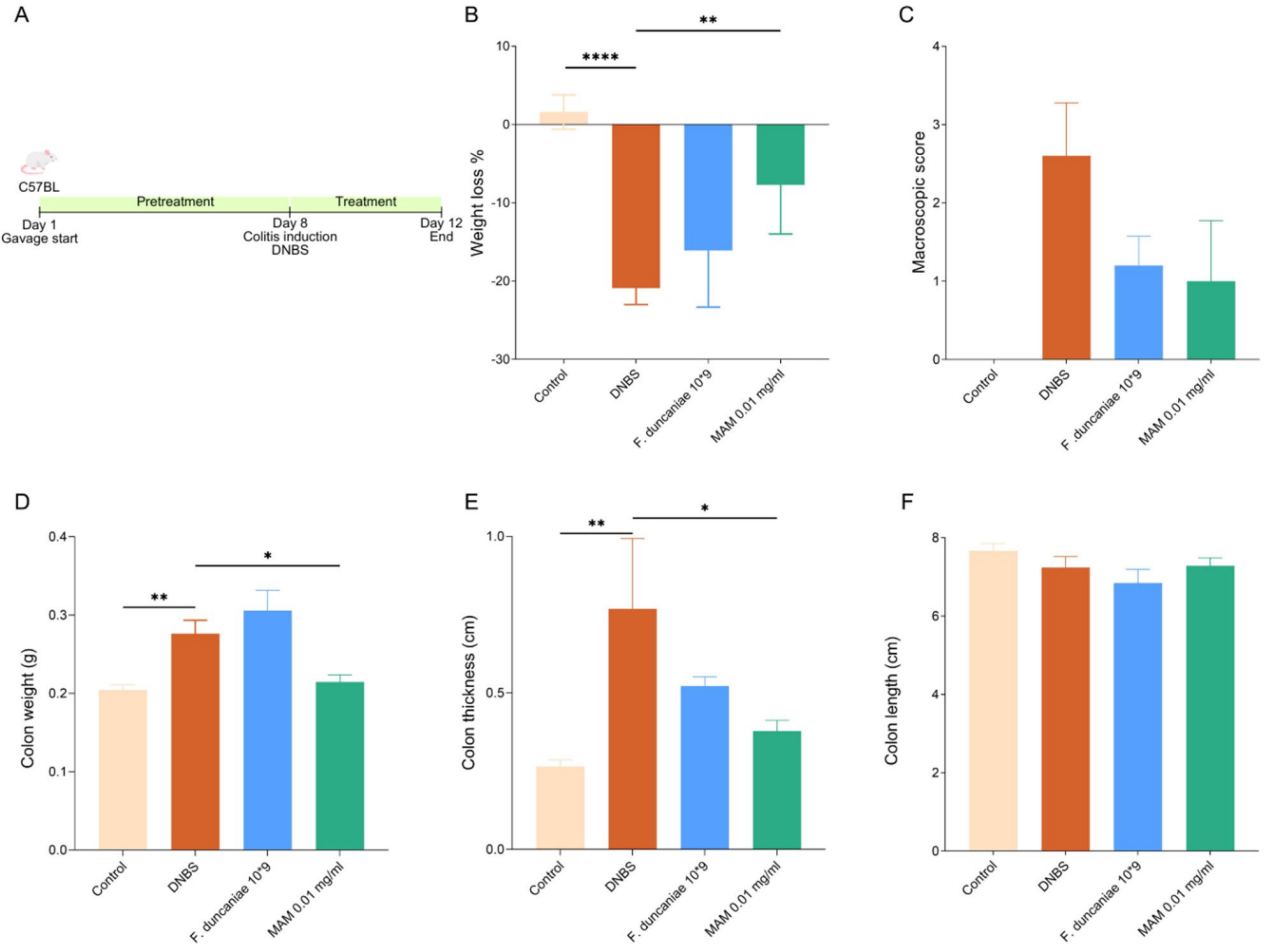
Effects of R-MAM in DNBS-induced colitis in mice. **a** Experimental design: Mice received oral R-MAM during the 12 days of the experiment. Colitis was induced by the intrarectal administration of DNBS on the 8th day. **b** Percentage of weight loss 96 h after DNBS administration. **c** Macroscopic score (*n* = 5–8 mice/group). **d** Colon weight (*n* = 5–8 mice/group). (**e**) Colon thickness (*n* = 5–8 mice/group). (**f**) Colon length (*n* = 5–8 mice/group). The orange bars represent the sick control, the blue bars represent the group treated with *F. duncaniae*, and the green bars represent the group that received R-MAM. Statistical analysis was performed via Dunnett’s test for weight loss, colon weight, and colon thickness. Dunn’s test was used for macroscopic scores. Significant comparisons are indicated: *p* < 0.0001 (****), *p* < 0.01 (**) and *p* < 0.05 (*)

After the in vitro studies, the effect of R-MAM was evaluated in vivo. The mice (*n* = 8 per group) were orally administered 1.0 mg/mL, 0.1 mg/mL, or 0.01 mg/mL R-MAM for 8 days as a pretreatment and 4 days following the intrarectal injection of DNBS to induce colitis (Fig. 4A). The higher doses (1.0 mg/mL and 0.1 mg/mL) did not significantly affect any of the analyzed parameters, including weight loss, macroscopic scores, or bowel measurements, compared with those of the sick control group (Supplementary Fig. 2). These findings suggest that higher concentrations may not confer additional benefits, in contrast with the therapeutic effects observed at lower doses. On the basis of these findings and previous in vitro analyses, we focused our investigation on the group that received the lower dose of R-MAM (0.01 mg/mL).

The weights of the mice were monitored during the 12 days of the experiment, and the mice were euthanized on day 12. Overall, R-MAM treatment (0.01 mg/mL) resulted in significant anti-inflammatory activity in vivo. R-MAM significantly protected mice from weight loss during DNBS-induced colitis compared with the sick control group (*p* = 0.0046) (Fig. 4B). While no significant differences were observed in the macroscopic disease scores, the scores were lower in the groups administered R-MAM. Furthermore, colon weight was significantly lower in the group treated with R-MAM than in the DNBS group, reaching levels similar to those of the healthy control group (*p* = 0.0301). A similar pattern was observed in the measurement of bowel thickness, where the thickness was significantly reduced in the R-MAM group (*p* = 0.0455), reinforcing the anti-inflammatory potential of R-MAM. No significant differences in colon length were detected between the treatment and control groups (Fig. 4C–F).

### R-MAM reduces neutrophil activity and pro-inflammatory cytokines in DNBS-induced colitis

**Fig. 5:**
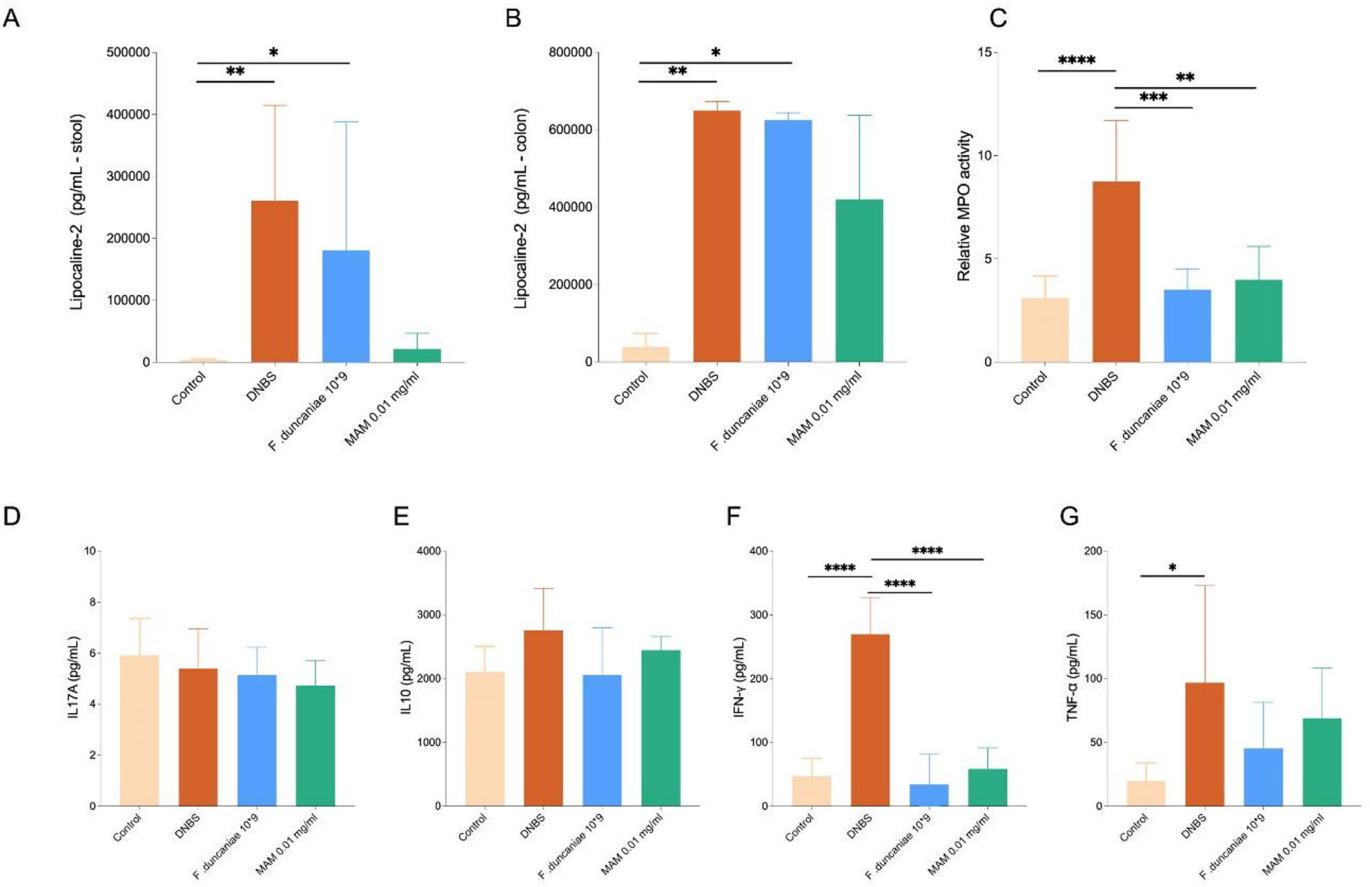
Quantification of inflammatory markers in the stool and colon of DNBS-treated mice. **a**, **b** Lipocalin-2 levels in stools and colon, respectively (*n* = 5 mice/group). **c** MPO activity (*n* = 5–8 mice/group). **D**–**G** Cytokine levels in colon homogenates: (**d**) IL-17 A, (**e**) IL-10, (**f**) IFNγ and (**g**) TNF-α (*n* = 5–8 mice per group). Statistical analysis was performed via ANOVA followed by Tukey’s post-test. Significant differences are indicated as follows: *p < 0.05, **p < 0.01, ***p < 0.001, ****p < 0.0001

To further characterize the anti-inflammatory effects of R-MAM, we quantified stool and colonic markers of inflammation. Lipocalin-2 concentrations were significantly increased in both stool and colon of DNBS-treated mice compared with controls (*p* < 0.01). Treatment with *F. duncaniae* or R-MAM did not significantly modify these levels, although a reduction in fecal Lipocalin-2 was observed in the group that received purified R-MAM (Fig. 5A, B). MPO activity was markedly elevated in DNBS mice (*p* < 0.0001) and was significantly reduced by both treatments (Fig. 5C). Analysis of colonic cytokines showed no significant changes in IL-17 A or IL-10 levels across groups (Fig. 5D, E). In contrast, IFNγ and TNF-α were strongly increased in DNBS-treated mice (*p* < 0.0001 and *p* < 0.05, respectively), and both markers were significantly reduced in the *F. duncaniae* and R-MAM groups (Fig. 5F, G).

## Discussion

*F. duncaniae* is one of the most abundant species in the gut microbiota, with the protein MAM identified as a key effector molecule mediating its beneficial effects, particularly in IBD [8, 17, 22, 29]. While earlier studies assessed MAM activity through cDNA delivery or eukaryotic expression systems [14, 17, 18], this study evaluated the direct effects of purified recombinant MAM in vitro using blood mononuclear cells and HT-29 cells, and on an intestinal colitis mouse model.

The direct exposure of TNF-α-stimulated HT-29 intestinal cells to R-MAM significantly reduced the secretion of the pro-inflammatory cytokine IL8. R-MAM at 0.05 and 0.1 mg/mL effectively decreased the IL8 level, with the 0.1 mg/mL concentration resulting in a statistically significant reduction compared with that in TNF-α-stimulated cells without R-MAM. Thus, these findings suggest that R-MAM can modulate immune signaling pathways in human intestinal cells associated with TNF-α-induced IL8 production and that a minimum effective dose of R-MAM is required to achieve anti-inflammatory effects.

Human mononuclear blood cells were also evaluated for their responsiveness to different doses of R-MAM without prior induction of inflammation, using LPS as a pro-inflammatory agent control. IL10 plays an important role in the intestinal anti-inflammatory pathway regulation by inducing Th17 responses and regulating macrophages that support intestinal barrier integrity [30–32]. Here, R-MAM triggered a dose-dependent increase in the secretion of the anti-inflammatory cytokine IL10. This finding aligns with previous reports highlighting the role of R-MAM in modulating immune responses via IL10 induction [22, 23].

TNF-α is a major driver of inflammation in IBD, but it also plays regulatory roles in immune priming and tissue repair [33, 34]. TNF-α is elevated in patients with intestinal inflammation, particularly in IBD, where maintaining a balanced TNF-α level is crucial for preserving gut homeostasis and preventing excessive inflammation [35, 36]. In this context, our results demonstrated that R-MAM also induces TNF-α production in a dose-dependent manner, with higher concentrations leading to higher TNF-α levels.

The IL10/TNF-α ratio was subsequently evaluated as an indicator of immune homeostasis, where a higher ratio suggests a shift toward an anti-inflammatory response, which is crucial for preventing excessive immune activation and tissue damage in the context of gut inflammation [37, 38]. R-MAM at a concentration of 0.1 mg/mL elicited a strong anti-inflammatory profile response, with IL10 levels approximately 14 times higher than those of TNF-α (*p* < 0.0001), suggesting that this concentration provides the most favorable anti-inflammatory profile.

In contrast, we also observed a concurrent increase in TNF-α at higher concentrations, particularly at 0.5 mg/mL. This raises questions about whether the observed TNF-α induction represents an exacerbated pro-inflammatory response or transient activation involved in immune priming and tissue repair. Given the dual role of TNF-α in inflammation and regulation, future studies should investigate the kinetics of cytokine production and assess whether prolonged exposure to high doses of R-MAM shifts the immune response toward a pathological inflammatory state or regulatory adaptation, raising questions about the balance between its pro- and anti-inflammatory effects [39].

With respect to the donor variations (healthy males and females aged 28–40), cytokine production among the four PBMC donors followed similar trends, although some donors presented higher cytokine levels. Except for donor 4, 0.1 mg/mL R-MAM consistently induced the highest IL10/TNF-α ratio, reinforcing its potential immunomodulatory role.

Since higher doses of purified R-MAM had no beneficial effects in vitro, we similarly observed that, among the three doses tested in vivo (0.5 mg/mL, 0.1 mg/mL, and 0.01 mg/mL), only the lowest dose (0.01 mg/mL) exhibited significant protective activity. This outcome may be attributed to immune overactivation, where excessive stimulation triggers exacerbated inflammatory responses instead of mitigating inflammation [40, 41]. Therefore, we focused on this dose to evaluate its anti-inflammatory potential in colitis.

Previous studies have evaluated MAM activity in intestinal models via eukaryotic expression systems or supernatants containing MAM peptides [21, 22]. While mRNA quantification confirmed MAM production in the gut, these indirect methods present challenges, such as the inability to control protein concentration precisely or assess its availability in the intestinal environment. In contrast, our study utilized purified R-MAM to directly evaluate its immunomodulatory activity, minimizing potential biases associated with delivery precision and accurate dose control, thereby addressing reproducibility concerns.

The administration of purified denatured R-MAM to DNBS-induced colitis model mice significantly ameliorated weight loss, colon weight, and colon thickness, which are well-established macroscopic markers of disease severity and inflammation in murine models [42]. Although the reduction in macroscopic disease scores was not statistically significant, the observed trend suggests a partial mitigation of inflammation and ulceration in R-MAM-treated mice. Similarly, no significant differences in colon length were detected between the groups, possibly reflecting the role of the early stage of colitis or R-MAM in preventing tissue remodeling.

In addition to the macroscopic improvements, R-MAM also modulated key biochemical and immunological markers of intestinal inflammation. Although lipocalin-2 levels, a commonly used marker of neutrophil recruitment and early mucosal inflammation, were not significantly reduced in either stool or colon, a decreasing trend was observed in the R-MAM group, suggesting a modest modulatory effect. More importantly, R-MAM decreased MPO activity, indicating a reduction in neutrophil infiltration and activation, which are critical components of acute colitis pathology [43]. Furthermore, cytokine analysis revealed that, while IL-10 and IL-17 A remained unchanged, TNF-α and IFNγ were significantly lower in R-MAM-treated animals. This pattern highlights the ability of R-MAM to attenuate central pro-inflammatory pathways without disrupting regulatory or Th17-associated responses. Interestingly, these in vivo findings partially diverge from our in vitro observations, where IL-10 secretion was strongly induced, suggesting that MAM-mediated immunomodulation may depend on the inflammatory environment and tissue context.

To date, the only study using purified recombinant MAM in vivo was conducted by Xu et al. in a type 2 diabetes mellitus (DM) model. They employed intragastric gavage, similar to our approach, but at a substantially higher daily dose of 200 µg of protein over 4 weeks [24]. In contrast, our highest tested dose (100 µg/day) had no protective effects, suggesting that the therapeutic window of MAM may vary depending on the disease model. Chronic metabolic inflammation in DM may require higher MAM concentrations to elicit beneficial effects, whereas acute inflammatory models such as DNBS-induced colitis may be more sensitive to immune modulation. These findings highlight the complexity of anti-inflammatory therapeutics and the importance of disease-specific responses.

Although the precise mechanisms underlying the effects of MAM remain unclear, its known properties suggest two potential modes of action: the modulation of anti-inflammatory responses and the restoration of mucosal barrier integrity. MAM’s ability to mitigate colitis symptoms by reducing the levels of pro-inflammatory cytokines, particularly TNF-α, was demonstrated here through in vitro studies and in the literature [21–23]. Additionally, MAMs were associated with the regulation of tight junction proteins, which are critical for maintaining epithelial integrity [24]. Such assays align with our observations of reduced weight loss and colon thickness in R-MAM-treated mice, suggesting that MAM supports gut barrier function and intestinal homeostasis.

Despite these important achievements, further analyses, including cytokine quantification, immune cell profiling, and histological evaluation, would provide deeper insights into the MAM’s mechanisms of action in intestinal inflammation [44]. Additional efforts with larger sample sizes and extended observation periods are also essential to validate and refine these findings, guiding preclinical trials in human models [45].

The purification of recombinant MAM was previously described by Xu et al. Similarly to our protocol, they used an *E. coli* expression vector to synthesize the protein, followed by purification through His-tag affinity chromatography [24]. Although these properties are beneficial, the authors did not provide further details regarding the stability of the purified protein. In the present study, R-MAM was probably not in its native conformation. This conclusion is supported by the presence of visible precipitates in the purified solution, indicating protein aggregation and potential misfolding under the applied purification conditions. This outcome was anticipated because of the hydrophobic nature of MAMs, which introduces challenges during purification, resulting in aggregation and potential misfolding [46]. Thus, the stability of purified R-MAM in the gastrointestinal tract remains a critical consideration of this work, as proteolytic degradation may limit its availability and activity in the intestinal environment. These structural alterations could partially impair its biological function by masking critical epitopes necessary for immune recognition and interactions with host molecules [47].

Despite this limitation, we proceeded with the experiments and observed significant anti-inflammatory activity, indicating the robustness of the effects of MAM, even under potentially suboptimal conditions. A possible explanation is that since R-MAM is likely processed in peptides via immune cells [48], its peptides retain anti-inflammatory activity. This hypothesis is supported by studies in which *F. duncaniae* supernatants containing MAM peptides were proven to be effective [19, 49]. Nevertheless, to increase the conformational stability of purified R-MAM and fully explore its therapeutic potential, future studies should consider optimizing purification methods. Strategies such as testing alternative expression systems, employing solubility enhancers, or coexpressing molecular chaperones during purification could be beneficial [50, 51].

Understanding these aspects is essential to elucidate the role and activity of MAMs in the host and unravel their full therapeutic potential. Thus, R-MAM holds potential as an adjuvant therapy alongside established IBD treatments, such as anti-TNF agents or immunosuppressants, potentially enhancing their efficacy while mitigating adverse effects. Additionally, its ability to modulate cytokine balance and support gut barrier integrity suggests that R-MAM could be developed as a postbiotic formulation, offering a safer and more stable alternative to live probiotics. Future studies should focus on optimizing its delivery, stability, and synergistic effects with existing therapies to explore and maximize its potential therapeutic applications in intestinal inflammatory diseases [52].

## Conclusions

Our study identified purified R-MAM as a novel effector molecule capable of stimulating anti-inflammatory responses in a dose-dependent manner, besides its protective effects observed in an in vivo intestinal colitis model. These results provide new opportunities for the use of R-MAM in the treatment of IBD and associated inflammatory diseases, although more research is needed to improve its formulation and investigate its mechanism of action and clinical uses.

## Supplementary Information

Below is the link to the electronic supplementary material.

Supplementary Material 1.

## Data Availability

All data generated or analysed during this study are included in this published article [and its supplementary information files].

## Abbreviations

MAM: Microbial anti-inflammatory molecule
DNBS: Di-nitrobenzene sulfate
DSS: Dextran sulfate sodium
IBD: Inflammatory bowel diseases
R-MAM: Recombinant MAM
IL10: Interleukin-10
NF-κB: Nuclear factor kappa B
IFNγ: Interferon-gamma
cDNA: Complementary DNA
MLN: Mesenteric lymph nodes
LB: Luria–Bertani
IPTG: Isopropyl β-d‐thiogalactoside
OD: Optical density
PBS: Phosphate-buffered saline
BCA: Bicinchoninic acid
DMEM: Dulbecco’s Modified Eagle Medium
PBMC: Peripheral blood mononuclear cells
ELISA: Enzyme linked immunosorbent assay
ZO-1: Zona occludens-1
LPS: Lipopolysaccharides
DM: Diabetes mellitus
