# Supplementary material for: Recombinant MAM from Faecalibacterium duncaniae exhibits a protective effect in DNBS-induced colitis
Source: Microb Cell Fact. 2025 Dec 6;25:6. doi: 10.1186/s12934-025-02877-9 (PMC12797504; doi:10.1186/s12934-025-02877-9)

**SUPPLEMENTARY FIGURES**

**1. Purified recombinant MAM following dialysis**



The figure demonstrates MAM under insoluble conditions, indicated by the presence of visible precipitates in the solution.

**2. Dose‑response evaluation of rMAM in DNBS‑induced colitis**

(A) Body weight loss, (B) macroscopic score, (C) colon weight, (D) colon thickness, and (E) colon length in mice treated orally with three rMAM doses (0.01, 0.1, and 1.0 mg/ml). Data are shown as mean ± SEM (n = 5–8). Statistical analysis: Dunnett’s test for weight loss, colon weight, and colon thickness; Dunn’s test for macroscopic scores. *p < 0.05, **p < 0.01, ****p < 0.0001.


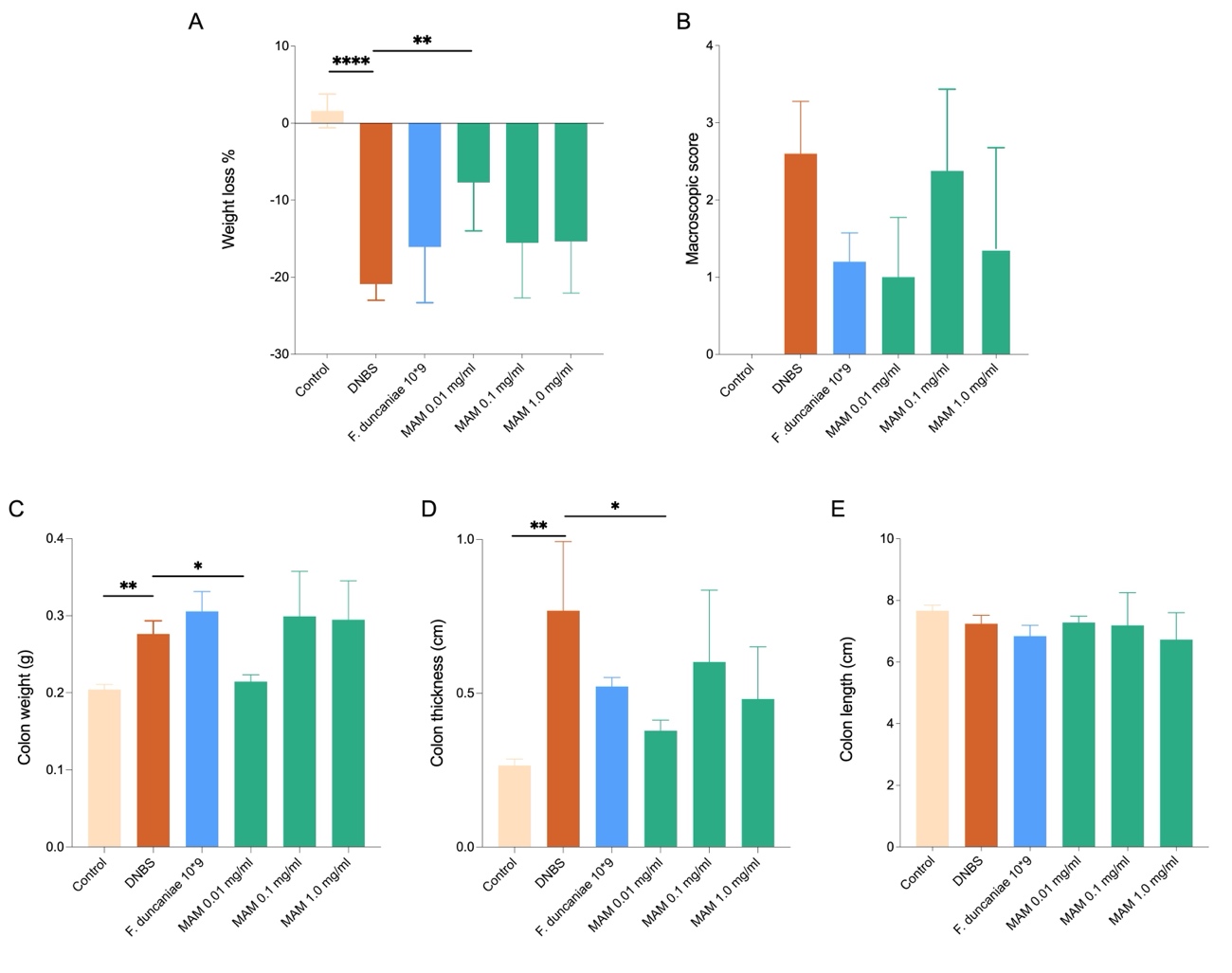

Supplement: Supplementary file 1 — Supplementary Material 1. [file 12934_2025_2877_MOESM1_ESM.docx]
